# A framework for retinal vasculature segmentation based on matched filters

**DOI:** 10.1186/s12938-015-0089-2

**Published:** 2015-10-24

**Authors:** Xianjing Meng, Yilong Yin, Gongping Yang, Zhe Han, Xiaowei Yan

**Affiliations:** School of Computer Science and Technology, Shandong University, 250101 Jinan, China; School of Computer Science and Technology, Shandong University of Finance and Economics, 250014 Jinan, China

**Keywords:** Improved gabor filter, Multi-directional multi-scale second derivation of Gaussian, Elongating filters, Retinal vasculature segmentation

## Abstract

**Background:**

Automatic fundus image processing plays a significant role in computer-assisted retinopathy diagnosis. As retinal vasculature is an important anatomical structure in ophthalmic images, recently, retinal vasculature segmentation has received considerable attention from researchers. A segmentation method usually consists of three steps: preprocessing, segmentation, post-processing. Most of the existing methods emphasize on the segmentation step. In our opinion, the vessels and background can be easily separable when suitable preprocessing exists.

**Methods:**

This paper represents a new matched filter-based vasculature segmentation method for 2-D retinal images. First of all, a raw segmentation is acquired by thresholding the images preprocessed using weighted improved circular gabor filter and multi-directional multi-scale second derivation of Gaussian. After that, the raw segmented image is fine-tuned by a set of novel elongating filters. Finally, we eliminate the speckle like regions and isolated pixels, most of which are non-vessel noises and miss-classified fovea or pathological regions.

**Results:**

The performance of the proposed method is examined on two popularly used benchmark databases: DRIVE and STARE. The accuracy values are 95.29 and 95.69 %, respectively, without a significant degradation of specificity and sensitivity.

**Conclusion:**

The performance of the proposed method is significantly better than almost all unsupervised methods, in addition, comparable to most of the existing supervised vasculature segmentation methods.

## Background

Retinal vasculature, which is the main structure visible in a fundus image, is the only non-traumatically observed part of the human circulation system. Many retinal diseases are characterized by changes to vasculature abnormalities, e.g., vessel dilation, tortuosity and presence of new blood vessels. And many system conditions, e.g., arteriosclerosis, hypertension or diabetes [1], can be diagnosed by evaluating the lesions of vasculature. Thus, the measurement of various vessels features, like width, branching patterns, tortuosity and texture can throw light on [2–4] early prevention of system disease and pathologies. However, manual extraction of these features is tedious and time-consuming, especially when facing complex vessel networks and large quantities of images. One acceptable way is to automatically measure the features, which has already aroused much attention in the medical world [5].

One of the optimal tasks is vasculature segmentation, which is a prerequisite step before the acquisition of several above-mentioned morphological features. Moreover, as an invariant feature, retinal vasculature is frequently used in retinal image registration in order to construct a global view of the eye fundus [6]. Besides, benchmark positions like fovea and optic disk can be located according to the tree-like topology of vasculature [7].Contrarily, in circumstances of detecting non-vasculature lesions, the vessels, as a kind of interference, must be excluded [8]. Finally, it is proved that the retinal vasculature is distinctive enough to be a new kind of biometric patterns, and several works have been published [9, 10].

Accurate vasculature segmentation has immediate and far-reaching impact to the above-mentioned applications. Yet segmenting the tree-like vessels is still a challenging task: (1) image level, existence of random noise and uneven illumination distributions; (2) vessel level, variability of vessel intensity, diameter and shape [11]; (3) pathological level, presence of vessel and non-vessel lesions bring large abnormal regions [12, 13]. Though retinal vessel segmentation have been long researched, there is still room for improvement.

In order to deal with the various challenges exist in vessel segmentation and at the same time keep the simplicity of the models to enable efficiency, we consider preprocessing as a critical step. Proper preprocessing operations can remove noises, enhance the contrast between vasculature and background and emphasize the intensity differences between lesions and vasculature. In addition, good preprocessing operations should not bring deformations to vasculature and should clarify the capillary vessels. If the vasculature are greatly enhanced, subsequent segmentation operations will also be facilitated.

Based on the above-mentioned consideration, a new matched filter-based vasculature segmentation method is proposed. The method consists of three main stages: In the first stage, we threshold the images processed using weighted improved circular gabor filter (ICGF) and multi-directional multi-scale second derivation of Gaussian (MMSDG). This operation is conducted on the observation that vessels and background can be easily separated when suitable preprocessing operations exist. The ICGF is a retinal image enhancement method which is first demonstrated in our previous work [14]. It can enhance the image contrast and at the same time clarify the capillary vessels. The MMSDG operation is also a novel technique to enhance vasculature structures. The images processed by MMSDG have more uniformly distributed backgrounds, which is complementary to IGCF. In the second stage, the raw segmented image is fine-tuned by a set of novel elongating filters. This operation preserves the true ridges and fills the hollow pixels of the raw segmented vasculature according to their various thicknesses, simultaneously with the noises significantly reduced. Finally, we eliminate the speckle like regions and isolated pixels, most of which are non-vessel noises and miss-classified fovea or pathological regions.

The proposed method is easy to understand because there is no complicated image understanding techniques adopted. The contrast of vasculature and the background is enhanced by weighted combination of ICGF and MMSDG, which simplified the subsequent segmentation operations. In our proposed method, the raw segmentation is a binarization by a single threshold, which can be fixed by pilot study. Despite its simplicity, this method is shown to be both efficient and effective for the retina segmentation task. The publicly available STARE [15] and DRIVE [16] databases which are commonly considered as benchmark databases for vasculature segmentation are used to measure the performance of our method. From the accuracy and the area under the receiver operating characteristic (ROC) curve (AUC), we can figure out that our approach outperforms almost all the unsupervised methods and most of the supervised methods.

## Related work

This section is a brief review of the existing techniques for vasculature segmentation in 2-D retinal images. The dedicate work of Fraz et al. [17] has presented a much more detailed survey of previous methodologies. In this paper, we divide the existing segmentation methods into two classes: unsupervised methods and supervised learning techniques.

### Unsupervised method

The unsupervised methods encode the human knowledge to identify the vasculature. Among these methods, features related to the intensity and structure properties are mainly considered. This kind of methods can be further subdivided into four major types: matched filter, vessel tracking, morphological processing and model based methods.

The matched filter based methods enhance the vessel features by convolving the retinal image with 2-D templates. The templates are designed to simulate designated vessel feature patterns and the convolution results will indicate the presence and saliency of the pattern. Matched filters generally work well, however, may fail when vasculature abnormalities and lesions exist and when meet the capillaries [18]. In the work of Chaudhuri et al. [19], the piece-wise linear segments are approximated by a Gaussian curve. pixels with highest responses are selected as vessels, and then post-processing is operated for final determination. Despite the Gaussian model [20], methods based on steerable filters [21] have also been proposed for its intrinsic advantages of faster computation. What’s more, this kind of filter works well on retinal image enhancement [22]. The technique presented in [15] combines local vessel attributes with region-based ones extracted from vessel structures, and allows for multiple branching. The seminal work of Gang et al. [23] presents a retinal vasculature detection method that involves 2nd order Gaussian filters with filters and thresholds changed adaptively.

The vessel tracking techniques consist of locating the center point of blood vessel segment and estimating next location, using the spatial distribution properties of vessels. The most prominent advantage of these method is their efficiency in computation and meaningfulness. With the vessel area highlighted, thorough scanning over the whole image which is usually very computationally intensive and requires more time and space is omitted. However, the tracking method may fail when random structures or noises exist. Moreover, vessels cannot be complete when vessels fade away or any bifurcation points are missed. Chutatape et al. [24] designed a tracking method which uses Gaussian and Kalman filters to detect blood vessels. This method at first originates seed points around the perimeter of the optic disc (OD). Then vessels are tracked using Kalman filtering according to the selected seeds. Branches are also important structures in vessel tracking, related techniques are also employed for vessel detection. Related tracking methods are adopted in [25].

Methods of mathematical morphology [26] are also introduced to vessel analysis. In [27], the authors design a vessel pattern detection method which combines morphological filters and cross curvature evaluation. In their method, the vessels are defined as bright patterns which are piecewise connected and locally linear. To differentiate vasculature from analogous disturbing patterns, the cross curvature evaluation is then performed. Clean linear structures are extracted using this method, but they are not always connected to each other. Fraz et al. [28] extract the vessel tree by combining vessel centerline detection and morphological bit plane slicing. Mathematical morphology have proved to be a proficient methodology for vessel detection. Another method [29] first extracts the vessel centerline by using differential filters, and then the vessels are filled by morphological operators. Still, when a vessel centerline is not correctly detected, the retinal vessels will be missed in the final segmented results.

Another kind of method is model based techniques which include models based on vessel profile [30], models based on active contour [13] and geometric models based on level sets [31, 32], etc.

### Supervised method

The supervised learning approaches learn the rules for vessel extraction utilizing a training set of manually segmented benchmark samples based on given features. The manually segmented blood segmented vessels which work as priori knowledge can guide the model training. On the other hand, careful vessel labeling and repeated training sample selection can improve the precision of segmentation. The supervised learning methods usually have superior performance than unsupervised methods, but some limitations still exist. The first limitation is that supervised method use various kind of features such as intensity, texture, etc. extracted from each pixel and its surroundings to find the vessels. The interrelationship of these features is complicate and the redundancy of these features always aggravates time and memory consumption. Another limitation is the need for labeled training data which has to be done by eye specialists or at least trained observers. For a typical retinal image, it will take an observer about 2 h on average for labeling [33]. Moreover, the labeled training data may vary according to different observers. The third limitation is that the supervised learning method is time-consuming because of the pixel-level processing.

In the work of Niemeijer et al. [34], for each pixel a feature vector which is extracted using multi-scale Gaussian and its derivatives is defined. Then the K-nearest neighbor (kNN) algorithm is employed to estimate whether a pixel is belong to vessels. Staal et al. [33] exploits the intrinsic property of vessel by using ridge profiles. In their method, 27 features are selected using a sequential forward selection method and a kNN algorithm is applied. In [35], features for each pixel are calculated by employing a multi-scale analysis using a Gabor wavelet transform. A Gaussian mixture model classifier is used to determine wether a pixel is a vessel pixel or not. Ricci and perfetti [11] apply a bunch of line operators to extract features, and then Support Vector Machine (SVM) is employed for classification. Lupascu et al. [36] introduce a supervised method which is named feature based Adaboost classifier (FABC) for vasculature segmentation. In their work, a 41-D feature vector extracted using different filters for each pixel has been defined. Neural Network has also been used in retinal vasculature segmentation [37, 38]. The methodology presented by Marin et al. [37] employs a 7-D feature vector consisted of moment invariant based and gray-level features. A multi-layer feed forward neural network is utilized for training and classification. The combination of the radial projection and the semi-supervised self-training method using SVM is employed by You et al. [39] for vessel segmentation. Moreover, in order to further improve performances, methods based on ensemble learning were employed to the segmentation of retinal vessel by Fraz et al. [40, 41].

## Methodology

**Fig. 1:**
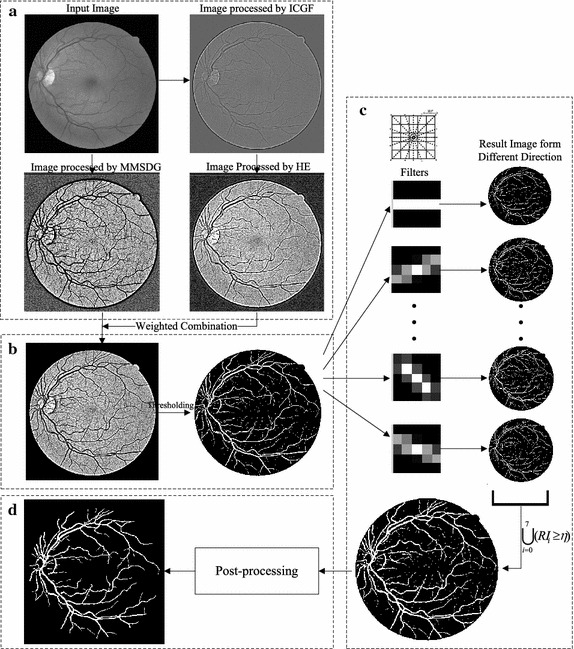
Functional diagram of the proposed method. **a** image enhancement; **b** preprocessing; **c** elongating filters; **d** post-processing

In this section, we begin with an overview of the proposed method. In order to be more comprehensible, a functional block diagram is used to systematically demonstrate the method, as shown in Fig. 1. Then each processing step in the proposed method is described in detail.

### Method overview

It is desirable to obtain segmentation methods for retinal vasculature which are both efficient and accurate. Unsupervised method plays an important role in vessel segmentation for its efficiency and effective utilization of pattern knowledge, thus attracts a great deal of interest worldwide. The four main steps in our segmentation algorithm are:

1. Enhancing the vessel structures and normalize the background distribution using weighted ICGF and MMSDG;
2. Coarsely segmenting the vasculature by assessing the proportion of vessels in the image;
3. Filtering the coarsely segmented region in eight different directions using a bank of filters (generated by rotating the vertical line using bilinear interpolation), then result image of different directions are intersected;
4. Removing the speckle-like regions and non-vessel pixels.

Each step of the proposed method is further illustrated in the following parts.

### Preprocessing

The green channel of the original color retinal image is selected in the proposed method, since the blood vessels in this channel have the highest contrast against the background. The blue channel tends to be empty and the red channel tends to be saturated. In addition, the retinal images always suffer from low contrast problems and random noise which can seriously affect the performance of segmentation algorithms. In our method, the retinal image is preprocessed using weighted ICGF and MMSDG.

#### Improved circular gabor filter

The ICGF works as a bias field removal operation. In [14], the additive bias is assumed by accumulating the retinal image with a template derived from Circular Gabor Filters [42, 43]. Let $Z_{r}(x,y)$ stands for the real part coefficient matrix of CGF and $Z_{i}(x,y)$ represents the imaginary part. The filtered image *R*(*x*, *y*) can be obtained by subtracting additive bias from the original image *I*(*x*, *y*):

1 $$\begin{aligned} R=I-\int \!\!\!\int I(x,y)(Z_{r}(x,y)-Z_{i}(x,y)i)\,dx\,dy \end{aligned}$$

Then the result image is normalized to 0 to 255 and further enhanced by histogram normalization. The final image is denoted as $Re^{\prime}$. The settings of the parameters are same as in [14].

#### Multi-directional multi-scale second derivation of Gaussian (MMSDG)

Blood vessels can be considered as dark elongated or line structures which are of different diameters and orientations on a brighter background. To make the vessels more salient and separable in terms of intensity, a preprocessing method based on enhanced vessels over different directions and scales is proposed. As vessels are of different diameters, different scales are used to calculate the various responses and then the maximum response at each pixel is kept.

In the proposed operation, the filtered image can be taken by convolving the image with derivatives of Gaussian using the Gaussian space techniques [44] as the way of how Hessian matrix is calculated, so the proposed method can be considered as an improvement over Hessian matrix at specified directions.

2 $$\begin{aligned} L_{x_{j}} = \frac{\partial {L(x,\sigma )}}{\partial {x_{j}}} = \frac{1}{2\pi \sigma ^2}\!\!\int _{x^{\prime}\in R^{2}}\!\frac{\partial {e^{-\Vert x-x^{\prime}\Vert ^{2}/2\sigma ^2}}}{\partial {x_{j}}}L(x^{\prime})\,dx^{\prime} \end{aligned}$$

In the equation, $x_{j}$ is the coordinate of image *L*(*x*, *y*) with respect to which derivative is taken. Mixed and higher order derivatives are computed by taking mixed and higher order of the Gaussian kernel. In the proposed method, the second order derivatives in *x* direction are taken as base filters.

3 $$\begin{aligned} L_{xx}=L\otimes \frac{\partial {^{2}G(x,y,\sigma )}}{\partial {x^{2}}} \end{aligned}$$

here, $L_{xx}$ is the second derivates of image *L*(*x*, *y*). The size of the second order derivation of Gaussian is set to $(6\sigma +1)\times (6\sigma +1)$.

**Fig. 2:**
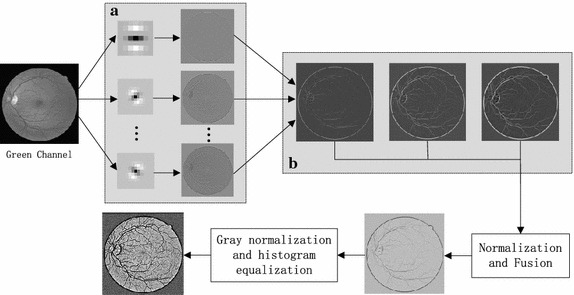
The flowchart of proposed MMSDG. **a** Multi-direction of the second derivation of Gaussian. **b** Filtered results in three different scales

The flowchart of the proposed MMSDG is demonstrated in Fig. 2.

The main steps in the MMSDG are as follows:

- Rotating the base filters into five different orientations using bilinear interpolation, thus filters of 0°, 30°, 60°, 90°, 120°, 150° are generated.
- Filtering the retinal image by convolving it with the generated filters, then, for each pixel, picking the maximum intensity value.
- Normalizing each image by dividing the sum of templates for each scale, respectively. Then, averaging the images from different scales.
- Normalizing the image into 0–255, and enhancing the image by histogram equalization.

#### Weighted ICGF and MMSDG

It is easy to find that images processed by ICGF and MMSDG can be complementary because ICGF considers the local intensity distribution according to each pixel in a fixed neighborhood area while MMSDG considers the binarized gradient globally. Such a complementary effect is shown in Fig. 1a, where the images processed by ICGF acquired clarified capillary vessel structures while those processed by MMSDG have uniformed background distributions. Combing ICGF and MMSDG we obtain the weighted image preprocessing method, as calculated in formula (4).

4 $$\begin{aligned} Res=\alpha I_{MMSDG}+(1-\alpha )Re^{\prime } \end{aligned}$$

where $0\le \alpha \le 1$ is used to control the relative contribution of ICGF and MMSDG. It is obvious that both ICGF and MMSDG are special cases of the weighted operation. The weighted method can generally work better than ICGF and MMSDG, and the parameter analysis experiment shows how the value of accuracy varies according to parameter $\alpha$.

### Coarse segmentation

After being preprocessed, the background of the retinal images is uniformly distributed with the vessel structures enhanced and clarified, which makes the segmentation possible by using a single threshold globally. In this part, we describe the simple idea of coarsely segmenting the vasculature by using the proportion of vessels in single images. The proportion can be obtained by statistically analyzing the datasets that used conventionally by researchers. In the experiment section, the proportion of the labeled vessels in the field of view (FOV) in each retinal database is described which is larger than proportion in the whole image. Suppose $\rho$ is the proportion of vessels against the whole image, *hist* is the intensity histogram of a single image, *hist*(*i*) denotes the *i*th value of *hist* which means there are *hist*(*i*) pixels are of intensity *i*. The global threshold $\theta$ can be defined as:

5 $$\begin{aligned} arg\quad min\left(\sum _{i=1}^{\theta }hist(i)\right)\ge \rho MN \quad (0\le i \le 255) \end{aligned}$$

where *M* and *N* stand for the height and width of an image, respectively. Then the image is binarized according to threshold $\theta$:

6 $$\begin{aligned} \!\! img(x,y)\!=\![img(x,y)\!\le \!\theta ] \quad (0\!\le \!x \!\le \! M, 0\!\le \! y \!\le \!N) \end{aligned}$$

The selection of proportion $\rho$ is not sophisticated since statistical analysis is available, but it is still essential because if $\rho$ is too small, capillary vessels will be ignored while if $\rho$ is too big, too much noise will be involved. The selection of $\rho$ is described in the experiment of parameter analysis.

### Elongating filters 

Retinal vasculature have tree-like structures which are locally continuous. Properly defined filters can remove noise, preserve the true ridge structures, and fill the hollow pixel in cortex vessel. Absent pixels can be viewed as anomalies in local ridges and that are the information we attempt to capture using a kind of elongating filters. In the proposed method, a group of filters with eight different directions are involved which are generated by rotated the vertical line using bilinear interpolation. The filters are in $5\times 5$ neighborhood. As shown in Fig. 1c. The scale of filters is also a compromise of vessel width and noises. If the filters are too large adjacent vasculature will be connected, while if the size is too small, the fine-tune effects turns to be invisible. Before filtering, the single pixels are eliminated since most of the single pixels are Gaussian noise. The coarse segmented images are then filtered and binarized before the intersection operation. Let $filt_{i}(0\le i \le 7)$ be the *i*th filter of the defined filter bank, $RI_{i}$ be the convolution result with $filt_{i}$. Then the filtered image can be defined as:

7 $$\begin{aligned} fimg=\bigcup _{i=0}^{7}(RI_{i}(x,y)\ge \eta ) \end{aligned}$$

here, $\eta$ stands for the threshold to binarize the group of filtered images. The value of parameter $\eta$ is also discussed in the experiment of parameter analysis, it can be settled based on observation, and modest change doesn’t affect the final performance abruptly.

### Post-processing

To obtain clean and accurate segmentation, another essential operation is post-processing. The image post-processing stage includes two basic procedures: denoising and speckle-like region removal. These two operations are implemented by measuring the region properties in an image. It is based on the observation that noises are always isolated regions and are discontinuous to each other. It consists of three steps: (1) determine the connected component; (2) compute the area of each component and acquire the length of major and minor axis of the ellipse that has the same normalized central moments as each connected component; (3) remove objects of small areas and speckle like components. The *regionprops* Matlab command [45] is adopted to implement the operation. When applied to the retinal images with speckle noises and vessels, it creates one structure for each component. Components that are too small in area are considered as noises and are eliminated from the image. And components that have equal major and minor axis length are most probably miss-segmented fovea or pathological regions. The effects of area threshold and ratio of major axis length divided by minor axis length are analyzed in the experiment section.

## Experiments

### Materials

The proposed method has been tested on the DRIVE [34]and STARE [15]databases which are publicly available and popularly used in vasculature segmentation evaluation. Both databases are aimed to convenient performance comparisons with existing segmentation methods and are considered as gold standard databases.

### Settings

All experiments for the proposed segmentation method are carried out with the following parameter settings, which were found with statistical analysis or pilot study.

In the MMSDG operation, six orientations are employed as described in the image enhancement sextion, and the parameter $\sigma$ has three values which is 1, 2 and 3. Thus three scales $7\times 7$, $13\times 13$ and $19\times 19$ are used. To best compromise the global intensity distribution and local contrast, the weight parameter $\alpha$ for ICGF and MMSDG is set to 0.33 for the STARE database and 0.43 for the DRIVE database.

The preprocessed retinal images are coarsely segmented with $\rho MN$ pixels labeled as vessels. In the STARE and DRIVE database, $0.081\times 565\times 584$ and $0.065\times 605\times 700$ are labeled as pixels belonging to vessels, respectively. The value of $\rho$ for the STARE database is smaller than value for the DRIVE database because images from the STARE database have large empty backgrounds. What’s more, with a different FOV, the vessels are not that rich as on the DRIVE database.

For the elongating filters, a threshold of $\eta =2.23$ is selected by pilot experiments to binarize the convolution results. If $\eta$ is too big, too much noise will be involve, and if $\eta$ is too small, even the vessels will be eliminated.

In the post-processing stage, components with an area that contains less than 40 pixels is eliminated. Objects with ratio of major axis length divided by minor axis length that is bigger than 0.3 and major axis length less than 40 pixels are eliminated.

### Performance measures

To facilitate comparisons with existing vessel segmentation methods, the proposed algorithm is evaluated using receiver operating characteristic (ROC) curves. In our work, the ROC curves are plotted as the threshold of the elongating filters varies. To be more clarifying, there are two kinds of pixels: vessel pixels and non-vessel pixels, consequently, there are four possible results according to whether each pixel is correctly classified, as shown in Table 1. In this paper, seven criterions are involved to evaluate the proposed method, which is shown in Table 2.

**Table 1:** Four possible results for vessel detection

	Vessel present	Vessel absent
Vessel detected	True positive (TP)	True negative (TN)
Vessel not detected	False positive (FP)	False negative (FN)

**Table 2:** Different criterions for performance evaluation

Measure	Description
Sensitivity (Sen)	TP/(TP + FN)
Specificity (Spe)	TN/(TN + FP) or 1 − FPF
PPV	TP/(TP + FP)
NPV	TN/(TN + FN)
Accuracy (ACC)	(TP + TN)/FOV pixel count
Accuracy (AUC)	Area under ROC curve
Kappa	(P(A) − P(E))/(1 − P(E))

The true positive fraction (TPF), also called sensitivity, is calculated as the rate of pixels correctly classified as vessel pixels (TP) divided by the total number of vessel pixels in the gold standard segmentation. The false positive fraction (FPF) in the number of pixels incorrectly classified as vessel pixels (FP) divided by the total number of non-vessel pixels in the gold standard segmentation. The positive predictive value (PPV) is the ratio of pixels classified as vessel pixels that are correctly classified. Negative predictive value (NPV) is the ratio of pixels classified as background that are correctly classified. The accuracy (Acc) for one image is the fraction of pixels correctly classified at a special threshold. As for the area under ROC curve (AUC), we can figure out that the closer a curve approaches the top left corner, the better the performance of the system. AUC is a single measure to quantify the behavior. We also compute the kappa values (a measure for observer agreement, where the two observers are the gold standard and the proposed segmentation method) [36].

### Results

#### Performance of the proposed method

In our experiment, the values of Sen, Spe, Ppv, Npv, Acc and Kappa were all computed according to pixels in FOV. The results are listed in Tables 3 and 4, respectively. The last rows of these tables show the average values of 20 images in each database.

**Table 3:** Performance results on DRIVE database images

Image	Sen	Spe	PPV	NPV	ACC	Kappa
1	0.7928	0.9770	0.8359	0.9697	0.9534	0.7871
2	0.7646	0.9893	0.9254	0.9604	0.9561	*0.8123*
3	0.6927	0.9859	0.8802	0.9555	0.9478	0.7462
4	0.7661	0.9830	0.8677	0.9665	0.9555	0.7886
5	0.7739	0.9854	0.8766	0.9703	0.9605	0.7999
6	0.6939	0.9853	0.8805	0.9537	0.9459	0.7459
7	0.7972	0.9764	0.7967	*0.9765*	0.9578	0.7734
8	0.7285	*0.9732*	*0.7461*	0.9707	0.9493	*0.7092*
9	0.7343	0.9791	0.8234	0.9653	0.9505	0.7486
10	0.7737	0.9823	0.8357	0.9739	*0.9606*	0.7816
11	0.7671	0.9839	0.8670	0.9686	0.9578	0.7903
12	0.7614	0.9806	0.8378	0.9689	0.9551	0.7726
13	*0.6780*	0.9858	0.8911	*0.9468*	*0.9406*	0.7367
14	0.7846	0.9757	0.7985	0.9737	0.9549	0.7662
15	0.7318	0.9768	0.7926	0.9677	0.9502	0.7333
16	0.7771	0.9807	0.8501	0.9690	0.9556	0.7869
17	*0.8041*	0.9736	0.7864	0.9762	0.9553	0.7700
18	0.7519	0.9868	0.8977	0.9628	0.9555	0.7932
19	0.6961	*0.9910*	*0.9287*	0.9509	0.9485	0.7670
20	0.7074	0.9832	0.8674	0.9559	0.9462	0.7490
Average	0.7489	0.9818	0.8493	0.9652	0.9529	0.7679

Italic fonts denote worst and cases in terms of each evaluation criterion

**Table 4:** Performance results on STARE database images

Image	Sen	Spe	PPV	NPV	ACC	Kappa
1	0.6313	0.9807	0.7999	0.9561	0.9427	0.6744
2	0.6580	0.9810	0.7769	0.9661	0.9515	0.6862
3	0.7702	0.9818	0.7900	0.9796	0.9645	0.7607
4	0.6295	0.9792	0.7754	0.9587	0.9435	0.6641
5	0.6161	0.9785	0.8011	0.9478	0.9339	*0.6601*
6	0.7376	0.9865	0.8405	0.9750	0.9646	0.7665
7	0.8418	0.9821	0.8529	0.9805	0.9667	0.8287
8	0.8301	0.9790	0.8182	0.9806	0.9638	0.8039
9	0.8326	0.9830	0.8553	0.9799	0.9669	0.8253
10	0.7173	0.9857	0.8614	0.9657	0.9561	0.7586
11	0.8381	*0.9750*	0.7838	0.9823	0.9616	0.7887
12	*0.8455*	0.9826	0.8516	0.9818	0.9681	*0.8307*
13	0.7469	0.9878	0.8951	0.9656	0.9584	0.7911
14	0.7451	*0.9898*	*0.9121*	0.9647	0.9593	0.7975
15	0.7146	0.9845	0.8604	0.9627	0.9527	0.7545
16	*0.6000*	0.9879	0.8900	*0.9381*	*0.9336*	0.6808
17	0.7817	0.9864	0.8896	0.9700	0.9613	0.8104
18	0.7978	0.9854	0.8032	0.9849	*0.9724*	0.7857
19	0.8360	0.9758	*0.6840*	*0.9896*	0.9675	0.7352
20	0.6562	0.9781	0.7509	0.9659	0.9487	0.6725
Average	0.7413	0.9825	0.8246	0.9698	0.9569	0.7538

Italic fonts denote worst and cases in terms of each evaluation criterion

**Fig. 3:**
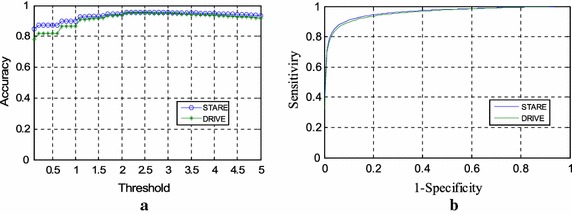
Performance figures. **a** Acc of the proposed algorithm varies according to the threshold parameter for DRIVE and STARE databases, respectively; **b** ROC curves for the two databases

As mentioned in the parameter setting section, the performance results shown in Tables 3 and 4 were obtained considering the same threshold value of $\eta$ for all images on the STARE database and DRIVE database, respectively. This value was set to obtain the maximum average accuracy (MAA) value. Figure 3a shows the MAA values calculated when the values of $\eta$ vary from 0.1 to 5 (step: 0.1) for the two databases, respectively. It is worth mentioning that the average accuracy values show no significant dependence on the value of $\eta$. The influence of parameter $\eta$ can also be reflected on the ROC curves for the two databases, as is shown in Fig. 3b. The MAA for the DRIVE and STARE databases are 0.9529 and 0.9569, respectively. The AUC calculated for the two databases under both curves are 0.9630 and 0.9739, respectively.

**Fig. 4:**
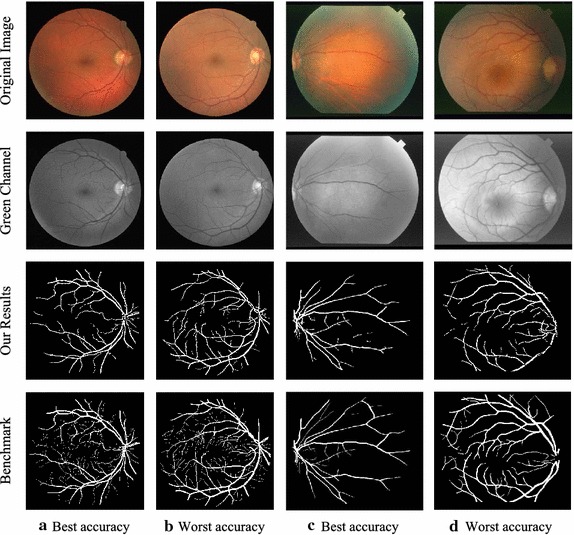
Best and worst accuracy cases for the DRIVE and STARE database. **a** Best accuracy case, image 10_test.tiff, accuracy 0.9606; **b** Worst accuracy case, image 13_test.tiff, accuracy 0.9406; **c** Best accuracy case, image im0291.ppm, accuracy 0.9724; **d** Worst accuracy case, image im0240.ppm, accuracy 0.9336

In Fig. 4, we display two segmented images along with their corresponding gold standard images and original images for each database. These segmented images are best accuracy and worst accuracy cases for each database.

The processing time of our algorithm is less than 3 s on the DRIVE database for a single retinal image on average, and less than 3.5 s on the STARE database, running on a PC with an Intel Core2Duo CPU at 3.2 GHz and 4GB of RAM. Since this method is experimented on MATLAB, which is an integrated platform, the performance might still be improved.

#### Comparison with existing methods

To facilitate comparison with other methods, sensitivity, specificity, maximum average accuracy and AUC were used as measures of method performance. The comparative methods listed in Tables 5 and 6 were selected from the most recent works—means that the criterion value is not answered in the original paper. From Table 5, we can see that the sensitivity, specificity, maximum average accuracy (MAA) and AUC on the DRIVE database are 0.7489, 0.9818, 0.9529 and 0.9664, respectively. The proposed method achieves the best performance among the unsupervised method, in addition, is superior to most of the supervised method. From Table 6, we can see that the sensitivity, specificity, MAA and AUC on the STARE database are 0.7413, 0.9825, 0.9569 and 0.9682, respectively. The specificity and accuracy of the proposed method achieve the best performance among both the unsupervised and supervised methods. The AUC is only second to the work of Lam et al. [12], since the sensitivity and specificity in their work is not answered, the sensitivity of the proposed method is only second to the work of Al-Diri et al. [13]. The work of Lam et al. achieves comparable performance due to its special consideration for lesions which can interfere vessel segmentation.

**Table 5:** Results comparison with existing methods on complete DRIVE database

No	Type	Methods	Year	Sen	Spe	ACC	AUC
1		2nd human observer	–	0.7796	0.9717	0.9470	–
2	Unsupervised methodology	Zana et al. [27]	2001	0.6971	–	0.9377	0.8984
3		Jiang et al. [18]	2003	–	–	0.9212	0.9114
4		Mendonca et al. [29]	2006	0.7344	0.9764	0.9452	–
5		Al-Diri et al. [13]	2009	0.7282	0.9551	–	–
6		Lam et al. [12]	2010	–	–	0.9472	0.9614
7		Miri et al. [26]	2011	0.7352	0.9795	0.9458	–
8		Fraz et al. [28]	2011	0.7152	0.9759	0.9430	–
9		You et al. [39]	2011	0.7410	0.9751	0.9434	–
10		Zhao et al. [32]	2014	0.7354	0.9789	0.9477	–
11		Proposed method	2015	*0.7489*	*0.9818*	*0.9529*	*0.9664*
12	Supervised methodology	Niemeijer et al. [34]	2004	–	–	0.9416	0.9294
13		Soares et al. [35]	2006	0.7332	0.9782	0.9461	0.9614
14		Staal et al. [33]	2004	–	–	0.9441	0.9520
15		Ricci et al. [11]	2007	–	–	*0.9595*	0.9558
16		Lupascu et al. [36]	2010	0.7200	–	*0.9597*	0.9561
17		Marin et al. [37]	2011	0.7067	0.9801	0.9452	0.9588
18		Fraz et al. [41]	2012	0.7406	0.9807	0.9480	*0.9747*
19		Vega et al. [38]	2015	0.7444	0.9600	0.9412	–

–, means the value is not answered in the reference paper

Italic numbers denote the best cases among unsupervised methods and the comparatively better results among supervised ones in terms each evaluation criterion

**Table 6:** Results comparison with existing methods on complete STARE database.

No	Type	Methods	Year	Sen	Spe	ACC	AUC
1		2nd human observer	–	0.8951	0.9384	0.9348	–
2	Unsupervised methodology	Hoover et al. [15]	2000	0.6747	0.9565	0.9264	–
3		Jiang et al. [18]	2003	–	–	0.9009	–
4		Mendonca et al. [29]	2006	0.6996	0.9730	0.9440	–
5		Lam et al. [30]	2008	–	–	0.9474	0.9392
6		Al-Diri et al. [13]	2009	*0.7521*	0.9681	–	–
7		Lam et al. [12]	2010	–	–	0.9567	*0.9739*
8		Fraz et al. [28]	2011	0.7311	0.9680	0.9442	–
9		You et al. [39]	2011	0.7260	0.9756	0.9497	–
10		Zhao et al. [32]	2014	0.7187	0.9767	0.9509	–
11		Proposed method	2015	0.7413	*0.9825*	*0.9569*	0.9682
12	Supervised methodology	Staal et al. [33]	2004	–	–	0.9516	0.9614
13		Soares et al. [35]	2006	0.7207	0.9747	0.9479	0.9671
14		Ricci et al. [11]	2007	–	–	*0.9584*	0.9602
15		Marin et al. [37]	2011	0.6944	0.9819	0.9526	*0.9769*
16		Fraz et al. [41]	2012	*0.7548*	0.9763	0.9534	*0.9768*
17		Vega et al. [38]	2015	0.7019	0.9671	0.9483	–

–, means the value is not answered in the reference paper

Italic numbers denote the best cases among unsupervised methods and the comparatively better results among supervised ones in terms each evaluation criterion

#### Parameter analysis

**Fig. 5:**
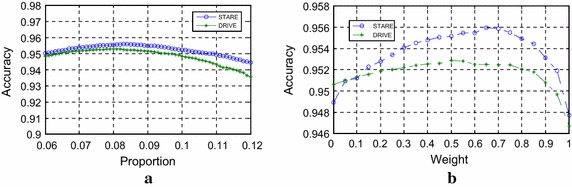
Parameter analysis. **a** Accuracy values when weight varies; **b** accuracy values when proportion varies

In this part, we discuss the influence of the parameter setting of $\alpha$ and $\rho$. In the weighted ICGF and MMSDG, the weight $\alpha$ is a tradeoff between the merits and shortcomings of these two operations. After being processed by the IGCF method, the image contrast is greatly enhanced and the capillary vessels are clarified. What’s more, another good property of the proposed IGCF is that regions around the vessels are brighter and noise free which makes the vessels even more separable. However, the IGCF only considers the local intensity distribution according to each pixel in a fixed neighborhood area. For the MMSDG, the second order deviations in multi-directions and multi-scales are considered, so the background distribution is better normalized with the vessels clarified. If $\alpha$ is too big, the result image of MMSDG holds a large proportion of the weighted image, although the background illumination is normalized, the noises around the vessels are too much. If is too small, it will be quite the opposite, the result image of the IGCF holds a large proportion of the result image, the noise around the vessels can be eliminated while the background distribution is not normalized enough. When $\alpha$ is set to 0.33, the best accuracy of the DRIVE database is achieved, when the weighted parameter $\alpha$ is set to 0.43, the best accuracy of the STARE database is acquired, as is shown in Fig. 5a. The accuracies are 0.9529 and 0.9569, respectively.

For the proportion of vessels picked in the coarse segmentation, it was observed statistically that an average of 12.7 % of retina pixels in the FOV is vessels [19]. In the coarse segmentation, the proportion is chosen to be smaller than the average proportion because the random noises must be considered. To better compare the effects of this parameter, *M* and *N* on the STARE database are set to 584 and 565, respectively. If $\rho$ is too big, too much noise is enrolled, and if $\rho$ is too small, too many pixels belong to vessel will be eliminated. $\rho$ is set to 0.081 for the DRIVE database and 0.083 for the STARE database when best accuracies are achieved,as is shown in Fig. 5b.

In the parameter analysis, only one parameter are changed each time. From Fig. 5, we can see that the performance changes slightly when parameter varies which implies robustness of the proposed method.

#### Component analysis

**Fig. 6:**
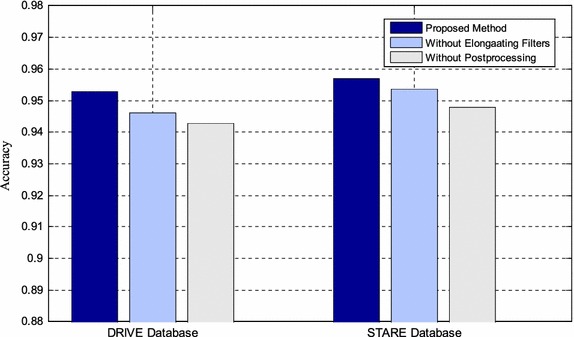
The necessity of functional components: elongating filters and post-processing

In this part, the necessity of the elongating filters and post-processing functional components from the proposed method is analyzed, as is shown in Fig. 6. This experiment is conducted by making each of the components absent. The parameter settings are same with the first experiment. The accuracies are 0.9460 and 0.9536 without elongating filters for the DRIVE and STARE databases, respectively. And accuracies without post-processing are 0.9429 and 0.9479 on the two databases, respectively. From the experimental results we can figure out that both the elongating filters and post-processing functional components are indispensable for the great degradation of accuracies. The degradations are 0.69 and 1 % on the DRIVE database and 0.3 and 0.9 % on the STARE database.

We also replaced the elongating filters with the morphological operations to show the superiority of the proposed operation over the traditional approaches. The accuracies are 0.9487 and 0.9548 on the DRIVE and STARE databases after replacing which have significant degradations compared with 0.9529 and 0.9569. The proposed method has better performances because the elongating filters treat the vasculature with various thicknesses adaptively. However, if the morphological operations are involved, all the vasculature are dilated and then eroded simultaneously, thus the spaces among bifurcations and isolated capillaries may be filled together with unnatural vasculature edges.

## Discussion and conclusion

The methodology represented in this paper was designed based on the observation that the vasculature can be easily separated if appropriate preprocessing operations exist. In the proposed method, the retinal images were first enhanced by weighted ICGF and MMSDG. After the preprocessing, the vessels were enhanced with the background uniformly distributed. What’s more, the true edges of the vessels were also kept. The enhancement simplifies the subsequent segmentation. The preprocessed images were then coarsely segmented by simple thresholding and fine-tuned by multi-directional elongating filters. Finally, the isolated pixels and speckle-like regions were eliminated. The proposed method were evaluated on the STARE and DRIVE database, the results reported in Tables 5 and 6 illustrate that, the performance of our method is superior to almost all the unsupervised method and most of the supervised method.

**Fig. 7:**
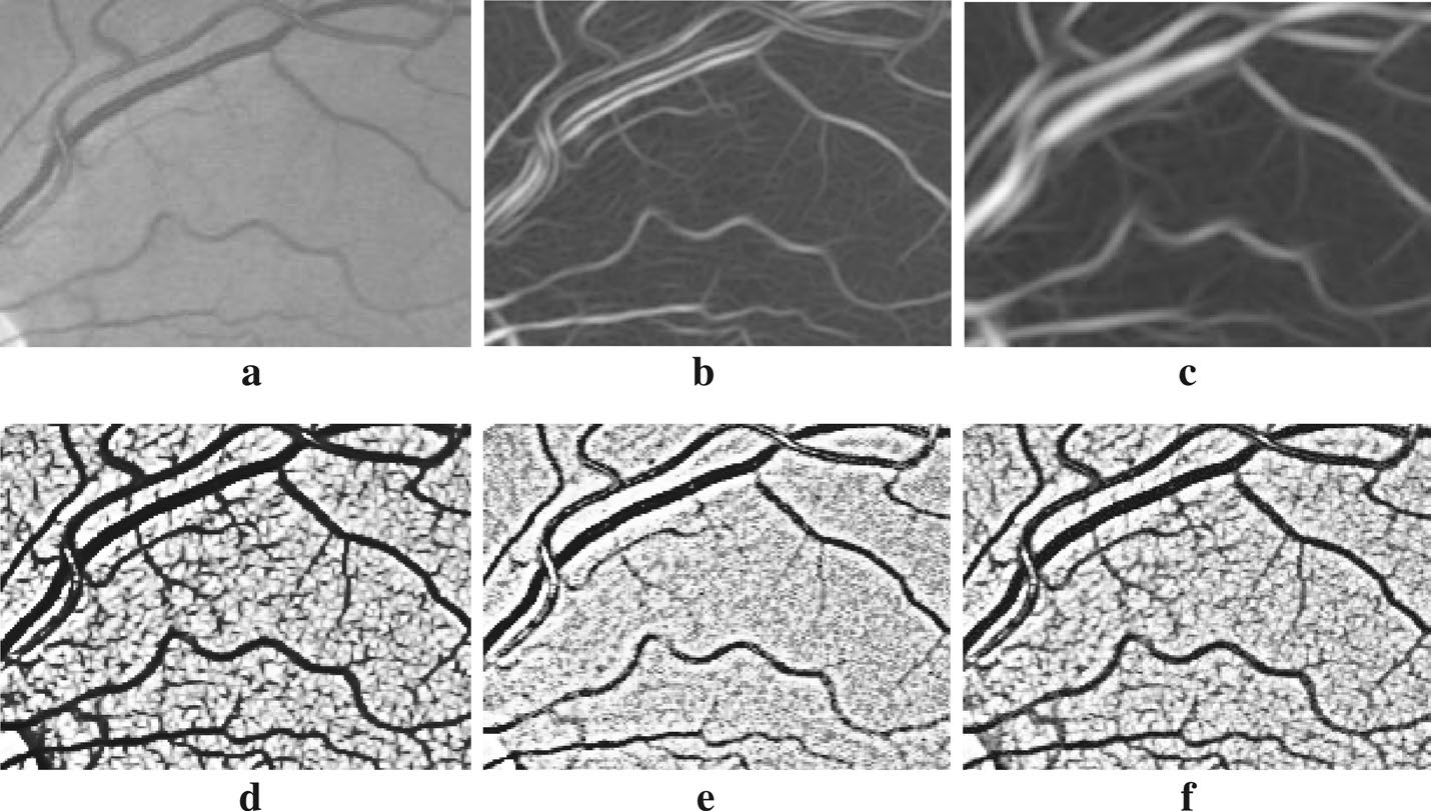
Preprocessing examples: a random patch from 01_test.tif. **a** Green channel, **b**, **c** two Gabor filter effects from [35], **d** processed by MMSDG, **e** processed by ICGF, **f** fusion effects

**Fig. 8:**
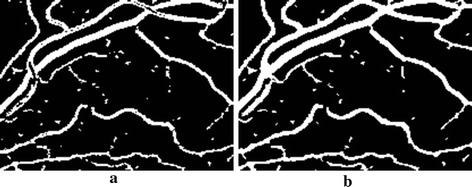
Effects after finetuning

The advantages of the proposed method lies in several aspects: (1) Non-deformation. Unlike most of the matched filter based methods (such as Gabor filters [35], Gaussian curves [21], etc.), which may cause vasculature deformations (Fig. 7b, c). The MMSDG method retains the high responses of second deviation of Gaussian from multiple directions and scales, thus the morphology characteristics are preserved (Fig. 7d). The ICGF operation also enhanced the vessels without deformations (Fig. 7e). (2) Edge enhancement. The ICGF operation normalized the background with the edges of vasculature greatly enhanced, which facilitates subsequent segmentation. Thus, the true edges of the vasculature are preserved, while most of the matched filter methods may result in images with blurry vessel edges (Fig. 7e, c). (3) Contrast stretching. By weighted combining MMSDG and ICGF, the background of the retinal images is uniformly distributed with noise significantly reduced. The vasculature are easily separable due to the high contrast to image backgrounds (Fig. 7f). (4) Adaptive fine-tunes. Unlike the basic morphological operations, the designed elongating filters fine-tune the raw segmented vasculature according to their various thicknesses, which bring better performances. The effects are shown in Fig. 8. In experiment of component analysis, we can also figure out that the fine-tuning operation is indispensable and if replaced by the morphological operators, the performances will be degraded. (5) Simplicity. Unlike most of the vessel tracking and model based methods, the proposed method doesn’t involve complicated image understanding and segmentation techniques. The proposed method is easy to understand and feasible to applications. (6) Robustness. In the experiments, we can figure out that the performances of the proposed method change slightly according to specific parameters. All the parameters involved can be fixed by pilot studies or common knowledge, which is convenient for untrained workers. (7) Efficiency. Tested on an integrated platform, the average processing time per image is less than 3.5 s, which means that the method represented in our paper is promising for real-time applications.

As a framework for vasculature segmentation, the generality of the proposed method can be seen in two aspects. First, the functional components can be used in other programs: (1) the retinal images enhanced by fused ICGF and MMSDG can be used as input of other vasculature segmentation methods. Moreover, clarified vessels and normalized backgrounds can also facilitate the performances of other segmentation models. (2) The fine-tuning and post-processing operations can be used in existing methods as additional processing steps. Second, each of the functional components of the framework can be replaced: (1) the raw segmentation operation is a simple threshold binarization, if more powerful segmentation models are involved, the performances of the proposed method may be improved. (2) The post-processing method can also be further improved or replaced by more powerful methods.

Though the proposed method achieves promising results, it still suffers from common problems of retinal vasculature segmentation. We prepared to make improvements in two aspects: Dealing with pathologies and Capillary vessels preserving.

In order to keep simplicity, we didn’t pay special attention to the cases with pathologies. Though we observed that the proposed method is relatively insensitive (from Table 7, we can figure out the superiority of the proposed method on the ten pathological STARE images) to abnormalities due to two reasons. First, the ICGF operation enhanced the vessels to lower intensities with the non-vessel pathologies like exudates and cotton wool regions to much higher intensities. Thus, the influences of non-vessel pathologies are suppressed. Second, vasculature pathologies like hemorrhages and aneurysms which are speckle like regions can be eliminated by the post-processing operations. Though the proposed method shows some advantages over both vessel and non-vessel pathologies, large scale hemorrhages and aneurysms connected to vessels are still hard cases. What’s more, the edges of non-vessel pathologies may give high responses which are also interferences to vessel segmentation, especially when overlapped with vessels. In our future work, the pathologies will be considered in the proposed work to father improve the performances.

**Table 7:** Performance comparison of results on pathological retinal images on STARE database

Methods	Sen	Spe	Acc
2nd human observer	0.8719	0.9384	0.9324
Hoover et al. [15]	0.6587	0.9565	0.9258
Soares et al. [35]	0.7181	0.9765	0.9500
Fraz et al. [41]	0.7262	0.9764	0.9511
Proposed method	0.7001	0.9823	0.9539

The ICGF operation has the ability to clarify the capillary vessels, but as the vessels fade away, the capillary vessels still tend to be discontinuous. Since the proposed method has been designed to be fast and provide high performances without understanding the image details, local vessel tracking was not involved. In the post-processing operation, the fragmented capillary vessels may also be eliminated. In the future, local tracking will be included to complete the vessel maps and to further improve the performance.
